# Erratum

**DOI:** 10.1590/0102-6720201700020020

**Published:** 2017

In the article “FROM COMPLEX EVOLVING TO SIMPLE: CURRENT REVISIONAL AND ENDOSCOPIC PROCEDURES FOLLOWING BARIATRIC SURGERY”, with the number of DOI: /10.1590/0102-6720201600S10031, published in the periodical Arquivos Brasileiros de Cirurgia Digestiva, 29 (Suppl 1): 128- 133, on page 128:

Where it read:

Received for publication: 02/16/2016

Accepted for publication: 6/2/2016

Read:

Received for publication: 8/16/2016

Accepted for publication: 02/10/2016

On page 132, it was added after completion:

ACKNOWLEDGMENTS:

This review has been published in part in the German language to disseminate the current topic to the public who speaks foreign languages ​​more widely.

On page 133, a reference number 66 was added:

66. Zorron R, Bothe C, Junghans T, Pratschke J, Benzing C, Krenzien F. [Conversational and endoscopic procedures after bariatric surgery]. Chirurg. 2016 out; 87 (10): 857-64. Doi: 10.1007 / s00104-016-0277-z. German. PubMed PMID: 27566189.

In the article “FOOD INTOLERANCES AND ASSOCIATED SYMPTOMS IN PATIENTS UNDERGOING FOBI-CAPELLA TECHNIQUE WITHOUT GASTRIC RING”, with the number of DOI: /10.1590/S0102-67202015000100010 published in the periodical Arquivos Brasileiros de Cirurgia Digestiva, 28 (1): 36- 39, on page 36:

Inclusion of author:

Cinthia Karla Rodrigues do Monte Guedes

In the article “HEREDITARY DIFFUSE GASTRIC CANCER: LAPAROSCOPIC SURGICAL APPROACH ASSOCIATED TO RARE MUTATTION OF CDH1 GENE151, on page 149:

Where it read:

Rafaella Higashi

Read:

Rafaella Ribas Muratori

In the article “IMMUNOLOGICAL EVALUATION OF PATIENTS WITH TYPE 2 DIABETES MELLITUS SUBMITTED TO METABOLIC SURGERY", with the number of DOI: /10.1590/S0102-6720201500040012 published in the periodical Arquivos Brasileiros de Cirurgia Digestiva, 28 (4): 266-269, page 266:

Where it read:

Financial source: none

Read:

Financial source: Foundation for Research Support of the State of Minas Gerais (FAPEMIG)

In the article “EFFECT OF THE INGESTION OF THE PALM OIL AND GLUTAMINE IN SERUM LEVELS OF GLP-1, PYY AND GLYCEMIA IN DIABETES MELLITUS TYPE 2 PATIENTS SUBMITTED TO METABOLIC SURGERY”, with the number of DOI: /10.1590/S0102-6720201400S100013 published no. Journal of the Brazilian Archives of Digestive Surgery, 27 (Suppl 1): 51-55, on page 51:

Where it read:

Financial source: none

Read:

Financial source: Foundation for Research Support of the State of Minas Gerais (FAPEMIG)

